# A Dual-Reconfigurable Printed Dipole MIMO Antenna with Frequency and Pattern Control for Mobile Devices

**DOI:** 10.3390/s26206529

**Published:** 2026-10-15

**Authors:** Mohammed A. Hassan, Ahmad H. Abdelgwad

**Affiliations:** 1 Electrical Engineering Department, College of Engineering, King Faisal University, Al-Ahsa 31982, Saudi Arabia; 2 Electrical Engineering Department, Fayoum University, Fayoum 63514, Egypt; aha05@fayoum.edu.eg

**Keywords:** handset antennas, MIMO, printed dipole, reconfigurable antenna, varactor diode

## Abstract

**Highlights:**

**What are the main findings?**

- A compact two-element MIMO dipole antenna is proposed with simultaneous frequency and radiation pattern reconfiguration using varactor-loaded driven and parasitic elements.
- The antenna achieves three tunable bands (2.2–2.8 GHz) and dual-beam steering (170° and 350°) with good agreement between simulations and measurements.

**What are the implications of the main findings?**

- The design offers a simple, low-cost, and efficient alternative to complex reconfigurable antennas by eliminating the need for elaborate switching networks or multiple control circuits.
- It enables practical integration into smart, cognitive radio, and next-generation wireless handheld devices requiring adaptive frequency and spatial diversity.

**Abstract:**

The design, analysis, and experimental development of a two-element reconfigurable printed dipole MIMO antenna are presented in this paper. The antenna exhibits both frequency and pattern reconfigurability and it is suitable for handheld devices. The frequency-tuning mechanism is implemented using varactor diodes mounted on the driven dipole elements while pattern reconfigurability is achieved using varactor diodes placed at the centers of parasitic dipoles, which are employed to steer the pattern. The fabricated antenna provides a relative frequency-tuning range of 24%, extending from 2.2 GHz to 2.8 GHz, within which the pattern can be reconfigured at 350° and 170° in the azimuth plane. The measurements validate the proposed concept, and the Diversity and MIMO system-level performance is investigated as well.

## 1. Introduction

Modern wireless communication systems are evolving toward reconfigurability to provide secure, reliable, and high data-rate communications. To obtain the optimal performance of smart wireless platforms, adaptive signal processing techniques as well as advanced hardware are required. Antennas are key enabling components that play an essential role in determining the overall performance of the wireless systems. Traditional antennas with fixed properties for specific functionalities severely limit the intelligence level that can be introduced into smart wireless systems. Consequently, reconfigurable antennas (RAs) [1] have recently emerged as a promising solution that can provide sufficient adaptability to meet the wireless system requirements in cost-effective, compact and light-weight packages. RAs have emerged as a promising solution for addressing several challenges associated with modern wireless communication systems, including spectrum scarcity, limited available space, increasing structural complexity, and stringent energy-efficiency requirements [2,3,4]. Although large-aperture and metasurface-based architectures offer considerable flexibility in beam manipulation, their implementation in handheld devices is constrained by the limited form factor and stringent requirements on hardware complexity, power consumption, fabrication cost, and biasing circuitry. These practical considerations have therefore motivated the development of compact and low-complexity beam-control techniques that can be readily integrated into the confined space of modern handheld terminals.

RAs can adapt their characteristics to achieve selectivity in operating frequency [5], radiation pattern [6], bandwidth [7], polarization [8], or a combination of these parameters [9,10,11,12,13], which can be realized using innovative combinations of circuit components and radiating structures. Three physical mechanisms can be employed to reconfigure the antenna parameters, using electrical devices such as PIN diodes [14], RF MEMS systems [15], or varactor diodes [16,17]; using mechanical changes [18]; and using material changes [19]. 

Frequency-reconfigurable antennas [14,15,16,17,18,19] are highly desirable because of their several distinct advantages in terms of bandwidth expansion, noise rejection and potential frequency agility that are useful for multi-frequency applications and wireless systems operating at different standards. However, with radiation pattern manipulation [6], the antenna can significantly improve the performance of wireless system, where it can be employed to improve security, avoid interference, and save energy by adjusting signal directions. In increasingly congested wireless environments, particularly within the 2.4 GHz WLAN and LTE Bands 7, 38, 40, and 41, frequency and radiation pattern reconfigurability can provide valuable degrees of freedom for interference management and spectral coexistence. Frequency agility enables the antenna to operate over less-interfered channels, while pattern reconfiguration provides spatial discrimination against undesired signals. These capabilities can potentially improve interference mitigation, link robustness, and overall communication reliability in shared-spectrum environments [20]. 

Frequency tuning is usually implemented by changing the effective length of the antenna using switches [14,15] or varactors [16,17], while pattern reconfiguration can be achieved by parasitic element tuning [6,21,22], phase shift integration into array elements [23], or using co-located radiating apertures [24]. Reconfigurable multi-input–multi-output (MIMO) [5,6] antennas can integrate the benefits of high data-rate capability and a great deal of flexibility; therefore, they are a promising candidate for new wireless systems. However, most prior designs rely on complex multi-layer or parasitic configurations with numerous switching components to achieve reconfigurability, which increases circuit complexity, biasing requirements, and overall size, limiting suitability for compact devices. In contrast, the proposed design combines simplicity, compactness, dual reconfigurability, and verified MIMO capability, advantages rarely achieved simultaneously, demonstrating its novelty and practical relevance for next-generation handheld wireless systems.

In this work, a new frequency- and pattern-reconfigurable MIMO antenna for handheld terminals is presented. It consists of two collinear radiating dipoles and two parasitic dipoles printed on two edges of a handset dielectric housing. Each element is loaded with a varactor diode. The varactors on the driven elements are used to tune the frequency while the varactors on the parasitic elements are employed to direct the pattern where they can be configured as reflectors or directors. Most existing research focuses on pattern reconfiguration at a single frequency; in contrast, the proposed antenna can provide simultaneous frequency and pattern reconfiguration. The operating frequency considered here is from 2.2 to 2.8 GHz for 2.4 GHz WLAN and LTE applications (bands 7, 38, 40 and 41). Nevertheless, utilizing the presented concept, much larger number of elements can be implemented at mm-wave frequencies that would be very attractive for many applications. Although the proposed concept can be extended to smaller wavelengths, varactor quality factor, parasitic loss, and reduced tuning efficiency at higher frequencies must be carefully considered, and suitable high-Q low-loss tuning technologies may be required for practical mm-wave implementations. 

The rest of this letter is organized as follows. Section 2 introduces the proposed antenna geometry and experimental demonstration. The simulated and measured results are presented in Section 3, followed by a state-of-the-art comparison with the related reconfigurable antennas in the literature in Section 4. The conclusions of the study are summarized in Section 5.

## 2. Reconfigurable Antenna Design

### 2.1. Proposed Antenna Structure and Operation

**Figure 1:**
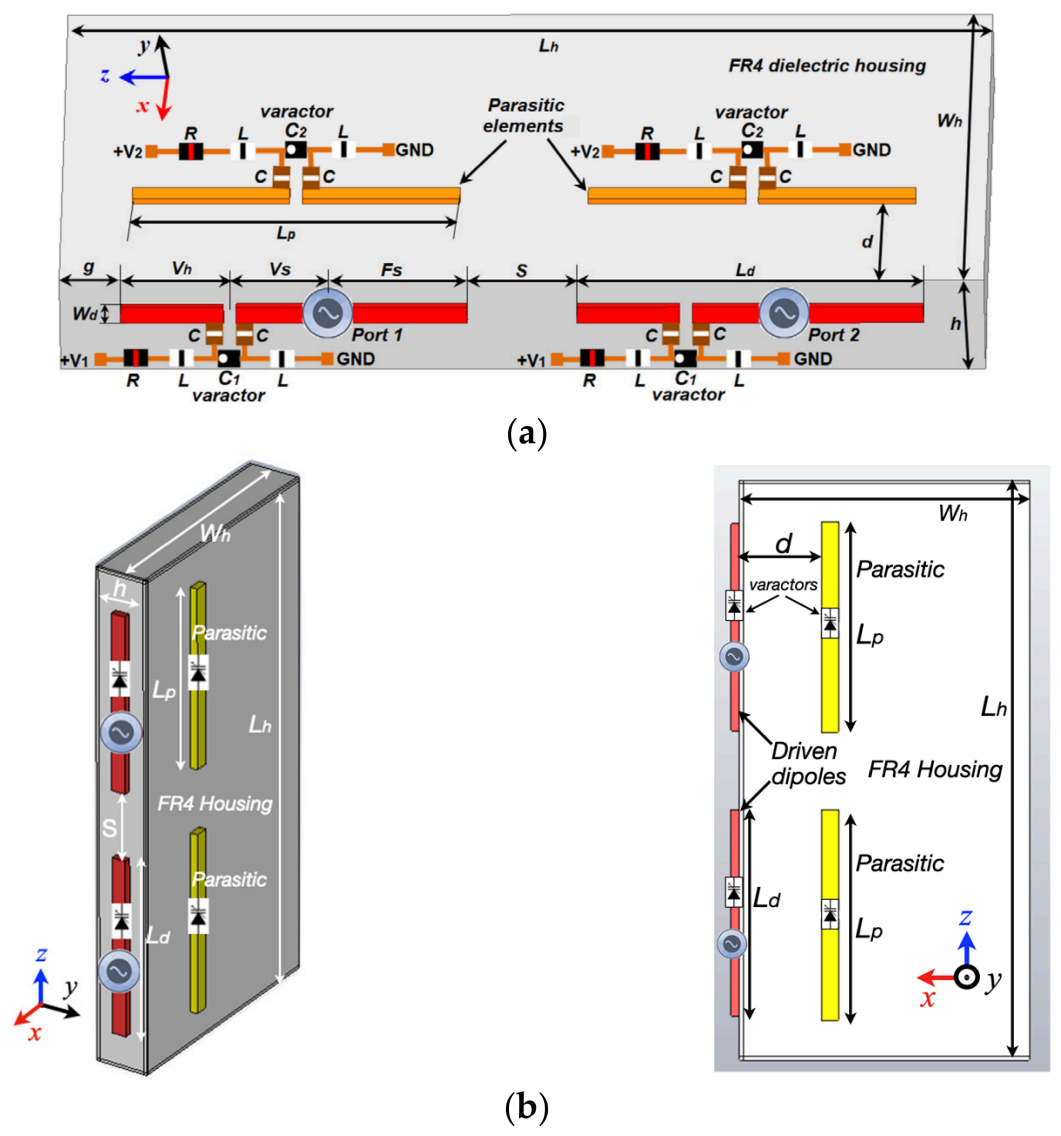
(**a**) Geometry of the proposed reconfigurable MIMO antenna; (**b**) 3D view and side view of the antenna design. Parameters: L_h_ = 140, W_h_ = 70, h = 10, L_d_ = 50, W_d_ = 2, S = 19, g = 10.5, F_s_ = 19, Vs = 11, V_h_ = 20, L_p_ = 51, d = 20 (all parameters are given in millimeters).

The geometry of the proposed antenna and its parameters is shown in Figure 1. As noted, there are two collinear driven dipoles printed along the narrow edge of a 0.8 mm thick FR4 (dielectric constant = 4.4, and loss tangent = 0.02) dielectric housing. Each dipole is loaded with a varactor diode to vary the effective length of each dipole through its equivalent capacitance (C_1_), thereby achieving frequency tuning. Furthermore, on the broad edge, a parasitic dipole element [25] is coupled to each driven one to control its pattern. By loading each parasitic dipole with a varactor, its effective length can be adjusted to act as either a reflector or a director. As introduced in [6,26,27], the voltages and currents of the driven and parasitic dipoles are related by the impedance matrix (Z):

(1) $$\left[\begin{array}{c} V_{d}\\ I_{p}X_{p} \end{array}\right]=\left[\begin{array}{cc} Z_{dd} & Z_{dp}\\ Z_{pd} & Z_{pp} \end{array}\right]\left[\begin{array}{c} I_{d}\\ I_{p} \end{array}\right]$$

where the subscript d and p correspond to the driven dipole and the parasitic dipole, respectively. $Z_{dd}$ and $Z_{pp}$ are the self-impedances while $Z_{dp}$ and $Z_{pd}$ are the mutual impedances. $V_{d}$ is the driven element excitation voltage and $X_{p}$ is the equivalent parasitic impedance. The coupling coefficient ($\alpha_{pd}$) can be calculated from (1) as given in (2):

(2) $$\alpha_{pd}=\frac{I_{p}}{I_{d}}=\frac{-Z_{pd}}{Z_{pp}-X_{p}}$$

The phase angle of the coupling coefficient determines if the parasitic dipole act as a reflector or director; it behaves as a reflector when the phase is positive and as a director when the phase is negative [28]. Evidently, the coupling coefficient strongly depends on the effective length of the parasitic dipole which can be controlled through the parasitic varactor capacitance (C2) to achieve pattern reconfiguration.

To electrically control the capacitances of the varactors, DC biasing circuits were also printed on the FR4 dielectric housing. Each circuit consists of a series of two RF chock inductors (L = 15 μH) and a current limiting resistor (R = 1.5 kΩ) that are connected to the dipole through two DC-blocking capacitors (C = 20 pF). Thus, the capacitances of the driven and parasitic varactors (C1 and C2, respectively) can be controlled through their corresponding applied reverse-bias voltages V1 and V2, respectively.

Although the reactive components in the biasing network (specifically the large 15 µH RF chokes and 1.5 kΩ resistors) introduce a time constant that limits the tuning speed to the microsecond or millisecond regime, this response time is highly adequate for the targeted WLAN and LTE applications. In such systems, reconfiguration is primarily utilized for band switching and mitigating slow-fading multipath environments, rather than rapid symbol-rate modulation.

Varactor diodes are integrated into the driven and parasitic elements, with their capacitances independently controlled by reverse-bias voltages V1 and V2, respectively. The voltage V1 tunes the driven-dipole electrical length and hence the resonant frequency, while V2 modifies the electrical length and coupling of the parasitic dipoles, allowing them to operate as either directors or reflectors. This independent control enables simultaneous frequency tuning and azimuthal beam steering within the proposed antenna configuration.

### 2.2. Experimental Demonstration

**Figure 2:**
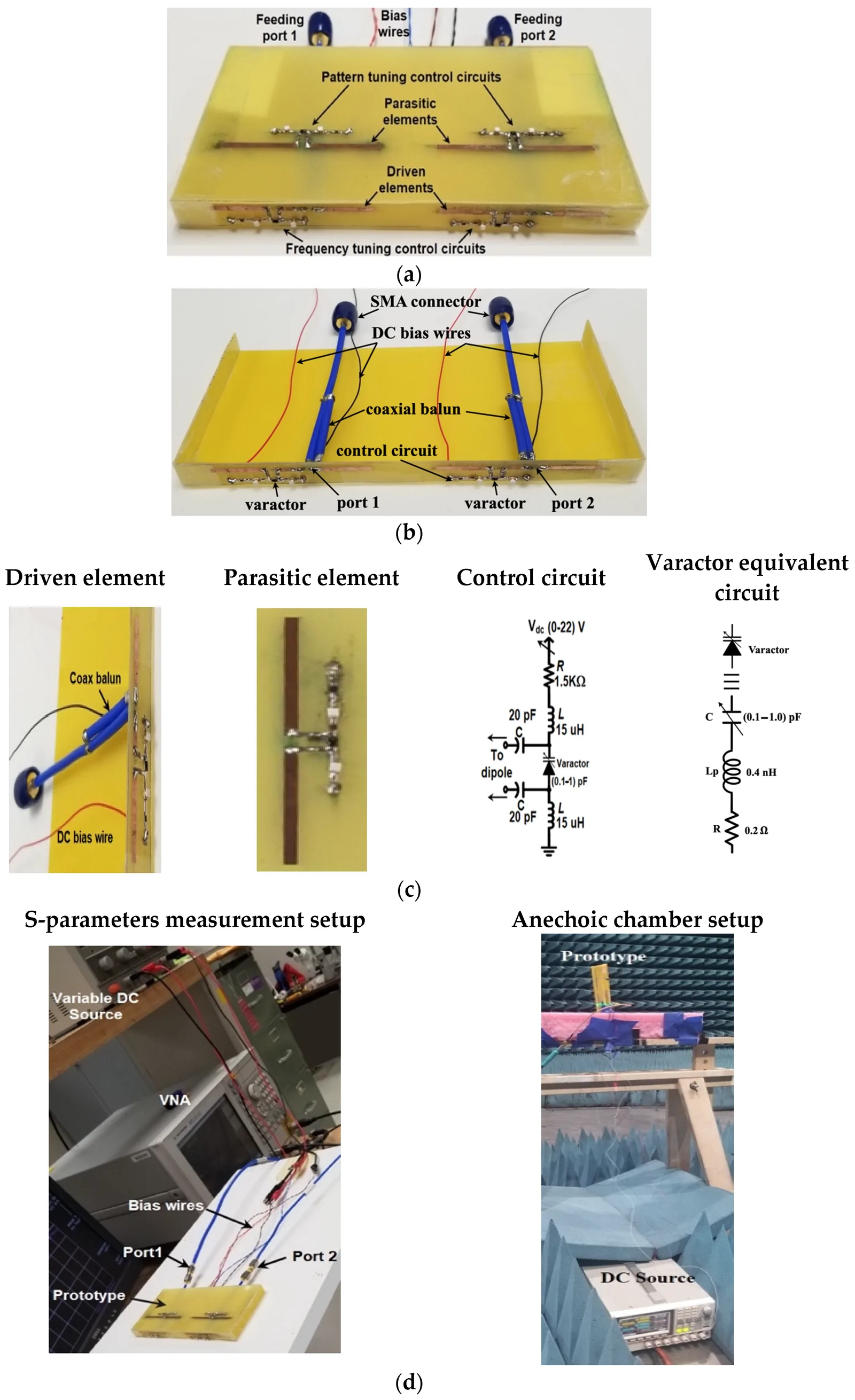
(**a**) Photograph of the built reconfigurable antenna prototype. (**b**) Antenna feeding and biasing network. (**c**) Antenna components. (**d**) S-parameters and chamber measurement setup.

A laboratory prototype of the proposed reconfigurable antenna was fabricated and measured to validate its operation. Photographs of the fabricated prototype and the measurement setup are shown in Figure 2. The driven elements and the parasitic elements were photoetched on separate FR4 PCBs, which were then attached together as shown in Figure 2a. Each driven dipole was fed using a 2.2 mm diameter semi-rigid 50 Ω coaxial cable and a split-coaxial balun fabricated from the same cable. All measurements were performed with the antenna elements fed through the coaxial balun to ensure consistent and balanced excitation of the driven dipoles.

The varactor equivalent capacitance employed in the simulation as well as applied voltage in measurements to achieve frequency and pattern reconfiguration are listed in Table 1. As shown, C_1_ (V_1_) was used to shift between frequency bands and C_2_ (V_2_) was used to switch between the reflector and director modes.

## 3. Simulated and Measured Results

The proposed antenna was designed and simulated using ANSYS HFSS (Version 21.2) Each driven dipole was fed with a 50 Ω source. Antenna tuning is facilitated by the MACOM MGV 125-20-0805 varactor diode (MACOM, Lowell, MA, USA), which is represented in the simulation environment as an equivalent series RLC circuit derived from the manufacturer’s datasheet as depicted in Figure 2c. It should be noted that the used MACOM varactor requires a relatively high reverse-bias voltage to provide the capacitance tuning range required for the demonstrated frequency and pattern reconfiguration. This voltage requirement is primarily associated with the characteristics of the selected tuning device and does not impose a fundamental limitation on the proposed antenna topology. For practical integration into commercial mobile platforms, the same reconfiguration concept can be implemented using alternative low-voltage varactors or other semiconductor tuning technologies offering a suitable capacitance range at lower control voltages. Such a substitution would enable compatibility with the low-voltage biasing levels commonly available in mobile electronics while preserving the independent control of the driven and parasitic elements. Therefore, the relatively high bias voltage used in the present prototype should be regarded as a characteristic of the selected proof-of-concept tuning device rather than an inherent limitation of the proposed antenna architecture.

The following subsections present the simulated and measured results of the antenna demonstrating its frequency and pattern reconfiguration capabilities as well as its MIMO performance.

### 3.1. Frequency Reconfiguration

**Figure 3:**
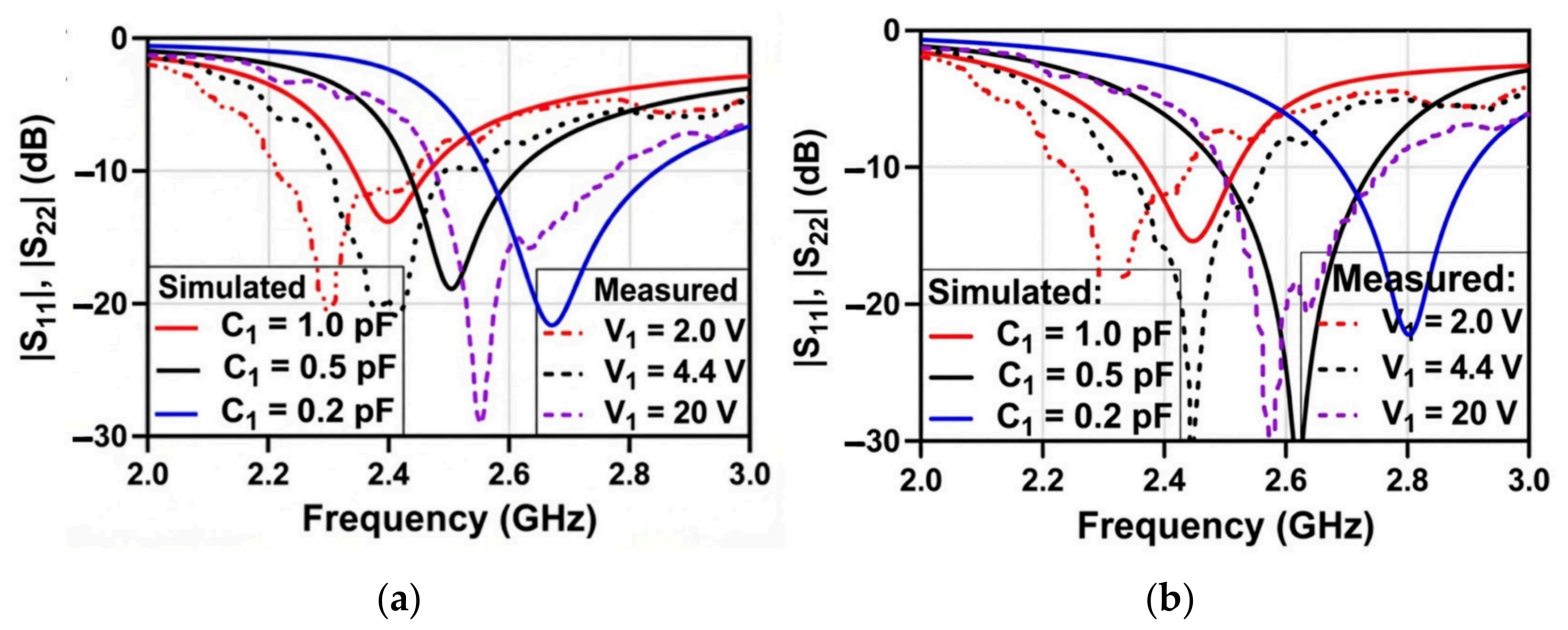
Simulated and measured reflection coefficients (in dB): (**a**) reflector mode; (**b**) director mode.

The proposed antenna was characterized in terms of its S-parameters. Measurements were performed using Agilent E5071C vector network analyzer (VNA). The system was calibrated using the standard two-port calibration procedure including short, open, load, and through connections. As the dipole elements are identical in structure, nearly identical results were obtained for both ports. The simulated and measured reflection coefficients for the reflector and director modes are shown in Figure 3. The simulated results show that the antenna can provide a wide tuning range from around 2.3 to 2.9 GHz (about 23%) for S_ii_ < −10 dB, with C_1_ varying from 0.2 to 1.0 pF. The secondary dips observed in some measured responses can be associated with the measurement/biasing structure, particularly the DC-bias lines and their interaction with the antenna and connectors. The measured resonant frequencies exhibit a slight downward shift relative to the simulated results, with a maximum deviation of approximately 100 MHz across the investigated tuning states. This discrepancy can be attributed to practical measurement and fabrication effects that are not fully accounted for in the simulation model. In particular, the SMA connectors, VNA cables, and DC-biasing wires introduce additional parasitic capacitance and inductance, which can alter the effective electrical length and consequently shift the resonant frequency. Furthermore, fabrication tolerances, variations in varactor mounting, and deviations in the actual dielectric properties of the FR4 substrate from their nominal values may contribute to the observed frequency differences. Nevertheless, the measured results exhibit consistent frequency-tuning trends with the simulations, demonstrating good agreement and validating the proposed reconfigurable antenna design.

**Figure 4:**
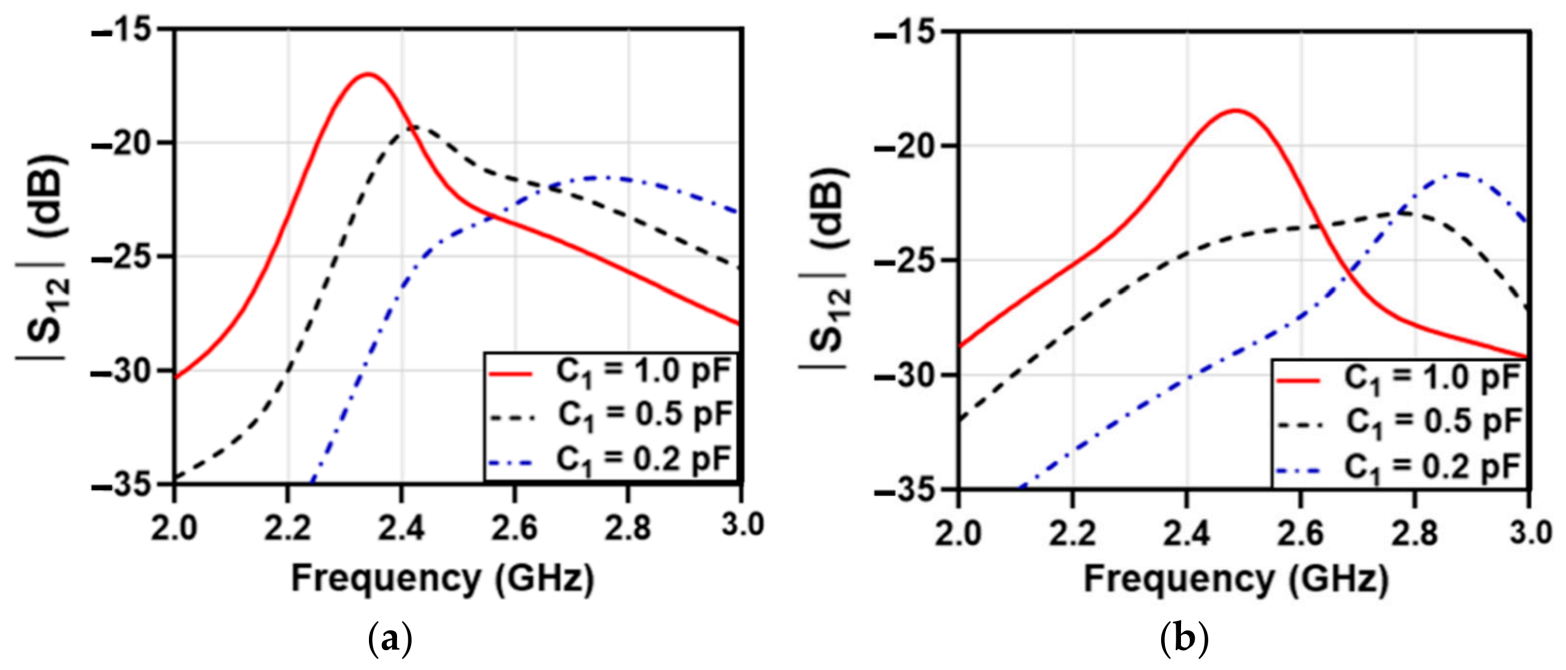
Simulated mutual coupling (in dB): (**a**) reflector mode; (**b**) director mode.

To ensure effective MIMO operation, low mutual coupling between the antenna elements is essential [29]. The simulated mutual coupling, represented by (S_12_), between the two antenna elements is presented in Figure 4. As observed, the proposed antenna achieves an isolation level better than 17 dB across the frequency bands of interest for the different reconfigurable states. Table 2 summarizes the maximum measured mutual coupling between the MIMO elements for the investigated frequency bands in both director and reflector configurations. The measured coupling remains below −15 dB for all operating modes and frequency bands, demonstrating satisfactory isolation between the antenna elements and validating the simulations.

The low mutual coupling can be attributed primarily to the collinear arrangement of the antenna elements, which helps reduce electromagnetic interaction between the radiating elements. In addition, the inter-element spacing (S) was carefully optimized to further suppress mutual coupling while maintaining the desired antenna dimensions and radiation characteristics. These design considerations contribute to the consistently high isolation achieved across the different reconfigurable operating states.

For integration into a mobile handset, metallic structures such as the PCB ground plane, battery, and LCD may influence the antenna characteristics by causing shifts in the resonant frequency, impedance, and radiation pattern. However, these effects can be minimized by maintaining an appropriate clearance around the driven and parasitic elements, particularly near the device edges. Moreover, the proposed varactor-based reconfiguration provides additional flexibility to compensate for moderate integration-induced variations by adjusting the effective electrical length of the driven elements as well as the coupling and relative phase of the parasitic elements.

**Figure 5:**
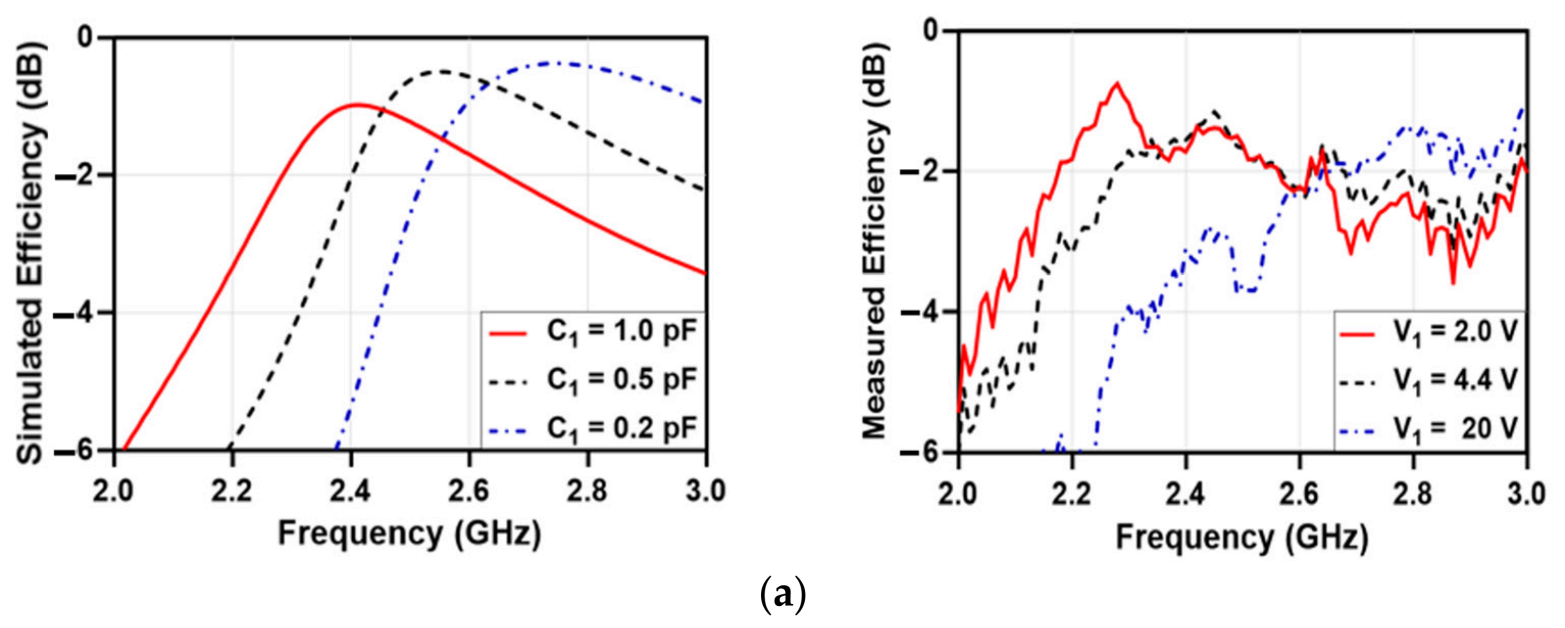

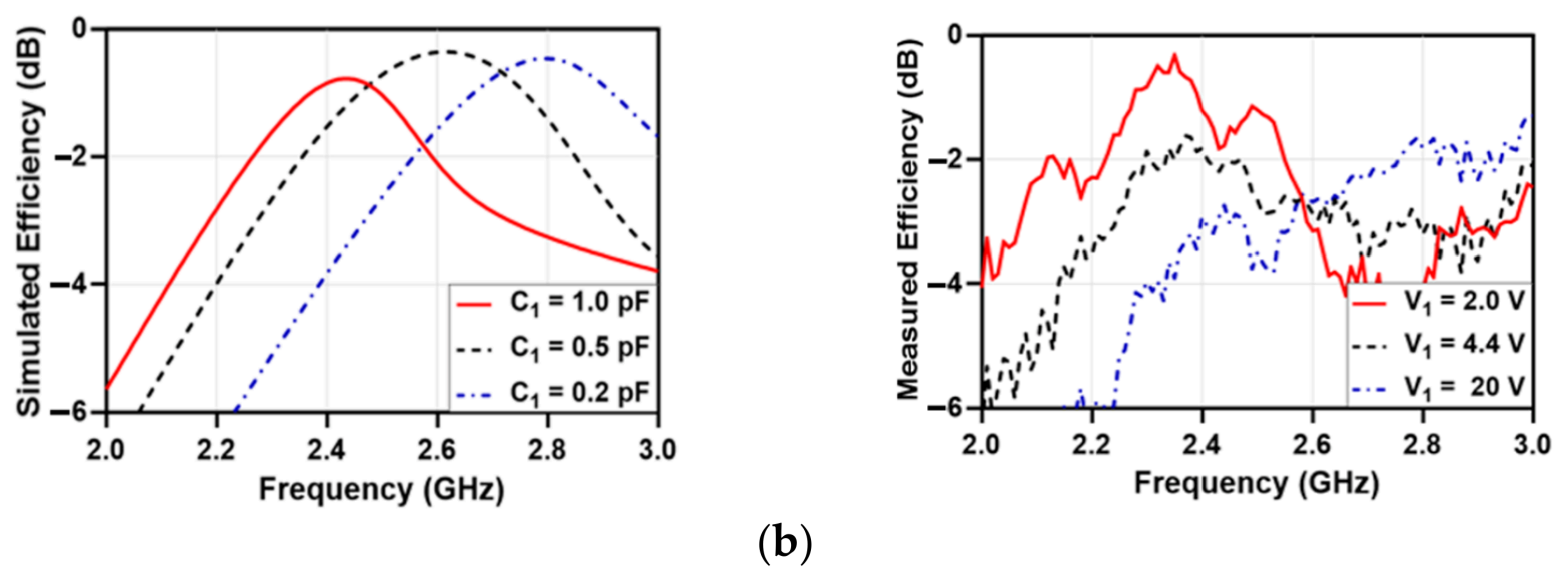
Efficiency (dB) for different capacitances of the driven-element varactor diode: (**a**) reflector mode; (**b**) director mode.

Figure 5 presents the simulated and measured element efficiencies, including mismatch and radiation components, for both reflector and director modes. With C_1_ tuning, the antenna maintains better than −2.0 dB efficiency over the targeted band. As observed in the measured results, the antenna exhibits a reduction in measured efficiency when tuned to the upper frequency bands above 2.4 GHz. To achieve resonance in this regime, a high reverse-bias voltage is required to drive the varactor diodes to a very low capacitance state (approaching 0.1 pF). Under these conditions, the equivalent series resistance (ESR) and inherent packaging parasitics of the diodes dominate the overall impedance. This effectively degrades the quality factor of the tuning network, inducing higher localized ohmic losses. Furthermore, tuning the antenna significantly above its fundamental physical resonance lowers its radiation resistance. Consequently, a larger fraction of the RF power is dissipated within the DC biasing network and the lossy FR4 substrate, rather than being radiated into free space. Despite these inherent tuning losses, the prototype maintains an acceptable efficiency of approximately 60% (−2.0 dB), which satisfies the requirements for practical mobile handset applications. This performance can be further enhanced in commercial applications by employing high-Q varactor diodes and replacing the standard FR4 material with a specialized low-loss RF substrate.

**Figure 6:**
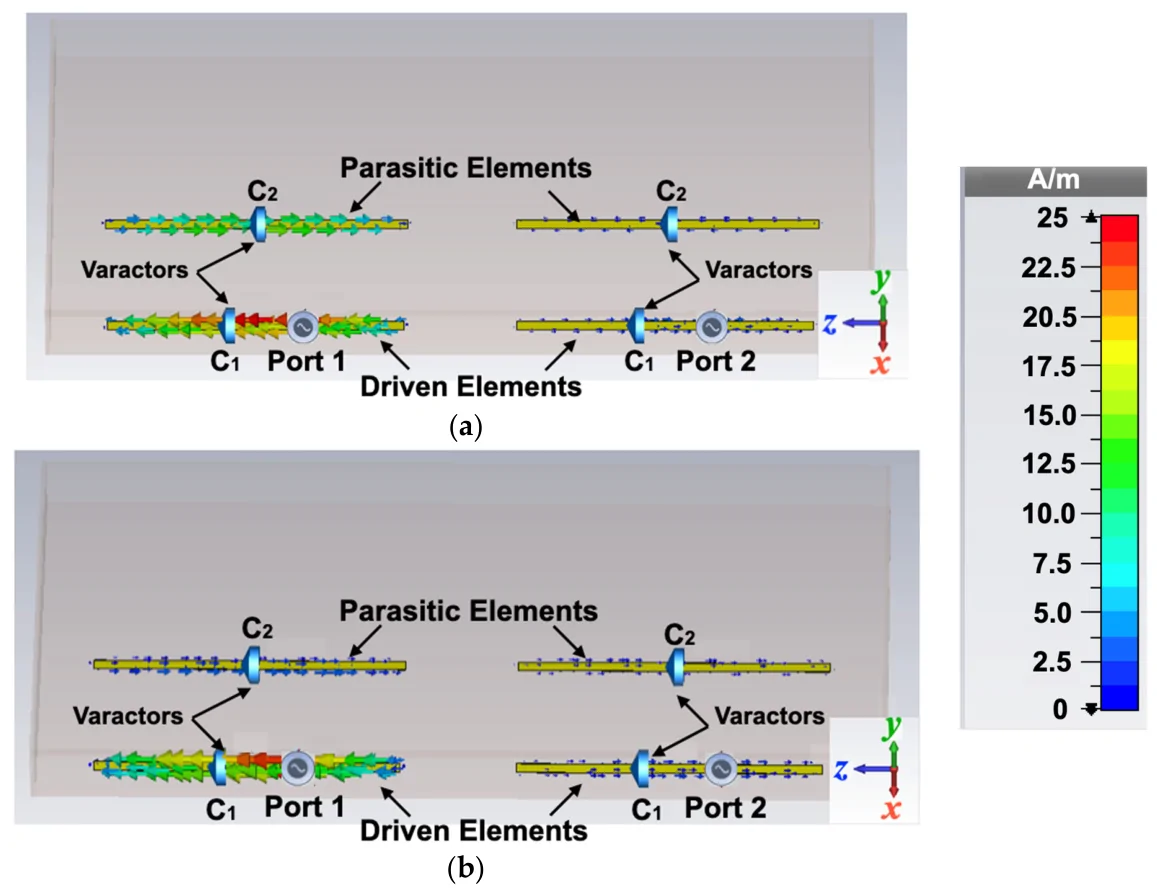
Simulated current distribution at 2.4 GHz: (**a**) reflector mode; (**b**) director mode.

Figure 6 presents the simulated surface current distributions at 2.4 GHz for the reflector and director operating modes, with Port 1 excited and Port 2 terminated with a (50 Ω) load. The current distribution along the driven element exhibits the characteristic behavior of a half-wavelength dipole, with maximum current density near its center and a gradual reduction toward the element ends. The mutual coupling between the driven and parasitic elements induces currents on the parasitic element, whose phase and amplitude determine the resulting radiation pattern. Accordingly, the main beam is directed toward the +x-direction in the reflector mode and toward the −x-direction in the director mode. The induced current on the parasitic element is stronger in the reflector mode than in the director mode, resulting in the observed difference in radiation characteristics. Moreover, owing to the high isolation between the two MIMO ports and the consequently weak inter-element coupling, only a negligible current is induced on the adjacent MIMO antenna element.

### 3.2. Pattern Reconfiguration

As discussed, each antenna element integrates a driven dipole coupled with a single parasitic element, forming a simplified version of a Yagi–Uda antenna configuration with a single parasitic element. The parasitic element, loaded with a varactor diode, can dynamically alter its electrical length and consequently its phase relationship with the driven element. Depending on this phase shift, the parasitic element behaves either as a reflector (positive phase) or a director (negative phase) [30].

**Figure 7:**
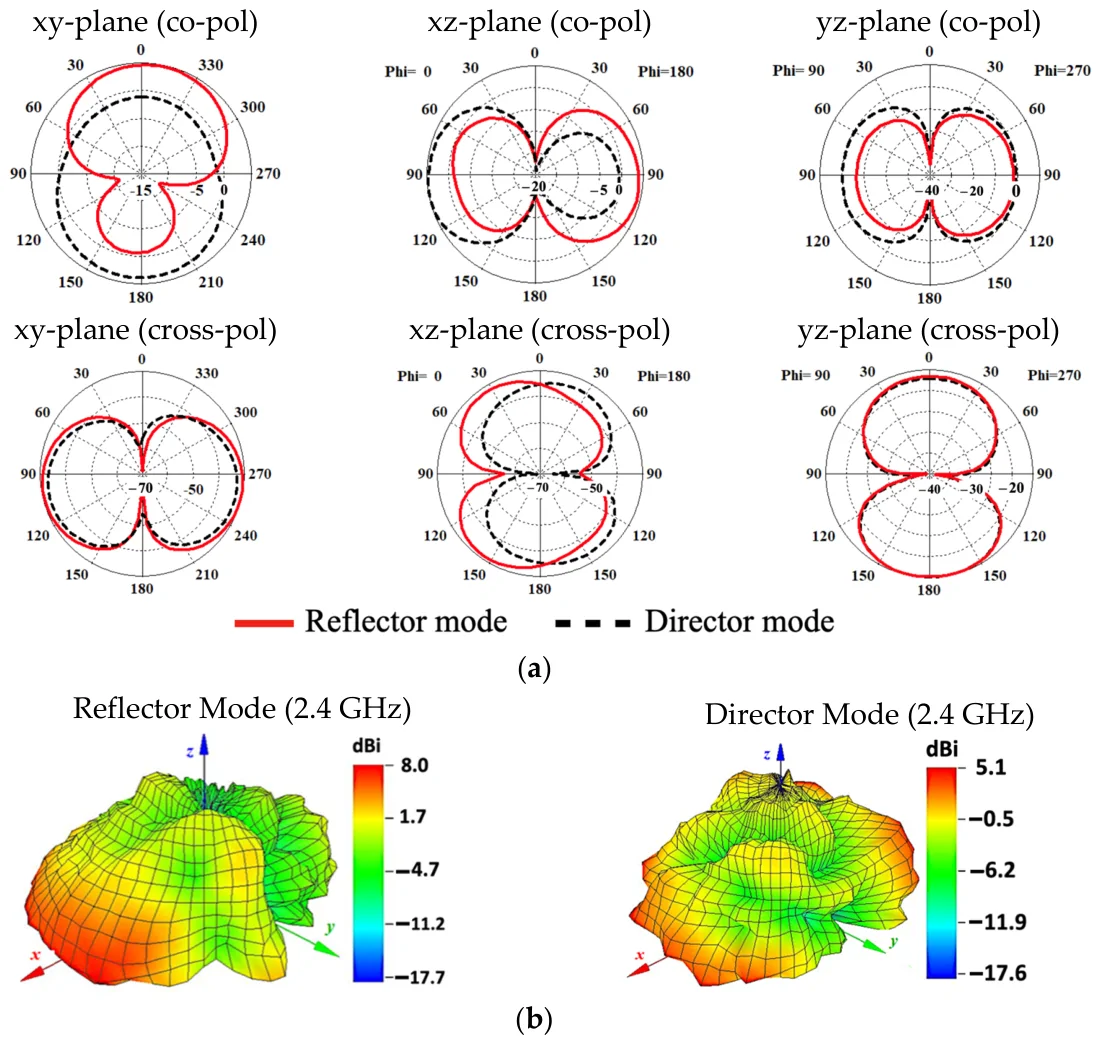
(**a**) Simulated azimuth-plane gain patterns (dBi) for the reflector and director modes at 2.4 GHz. (**b**) Measured 3D gain pattern (dBi) for reflector and director mode.

The simulated 2D gain patterns at 2.4 GHz for the principal xy-, xz-, and yz-planes are illustrated in Figure 7a for both the reflector and director modes, which are configured by adjusting C_2_. As evident from the results, varying the varactor capacitance C_2_ enables beam steering in two opposite azimuthal directions. For the azimuth xy-plane, when operating in the reflector mode, the primary radiation beam points toward ϕ = 350°, while switching to the director mode redirects the beam to ϕ = 170°. This minor angular deviation, approximately 10° off the absolute *x*-axis (ϕ = 0° and ϕ = 180°), arises directly from the physical geometry of the antenna. Specifically, the driven elements are positioned on the edge of the handset housing, whereas the parasitic elements are located on the side of the dielectric housing. Because these components reside on separate planes, steering the radiation pattern either toward the parasitic structure (director state) or away from it (reflector state) inherently introduces this slight tilt in the main beam direction.

Figure 7b illustrates the measured 3D gain patterns at 2.4 GHz for the upper hemisphere (θ = 0° to 90°). Due to the structural symmetry of the antenna across the xy-plane, the radiation characteristics in the lower hemisphere (θ = 90° to 180°) are mirror-identical. As the varactor bias voltage increases, the parasitic element transitions from a reflector to a director, effectively steering the main beam within the horizontal plane (θ = 90°) from the +x- to the −x-direction.

**Figure 8:**
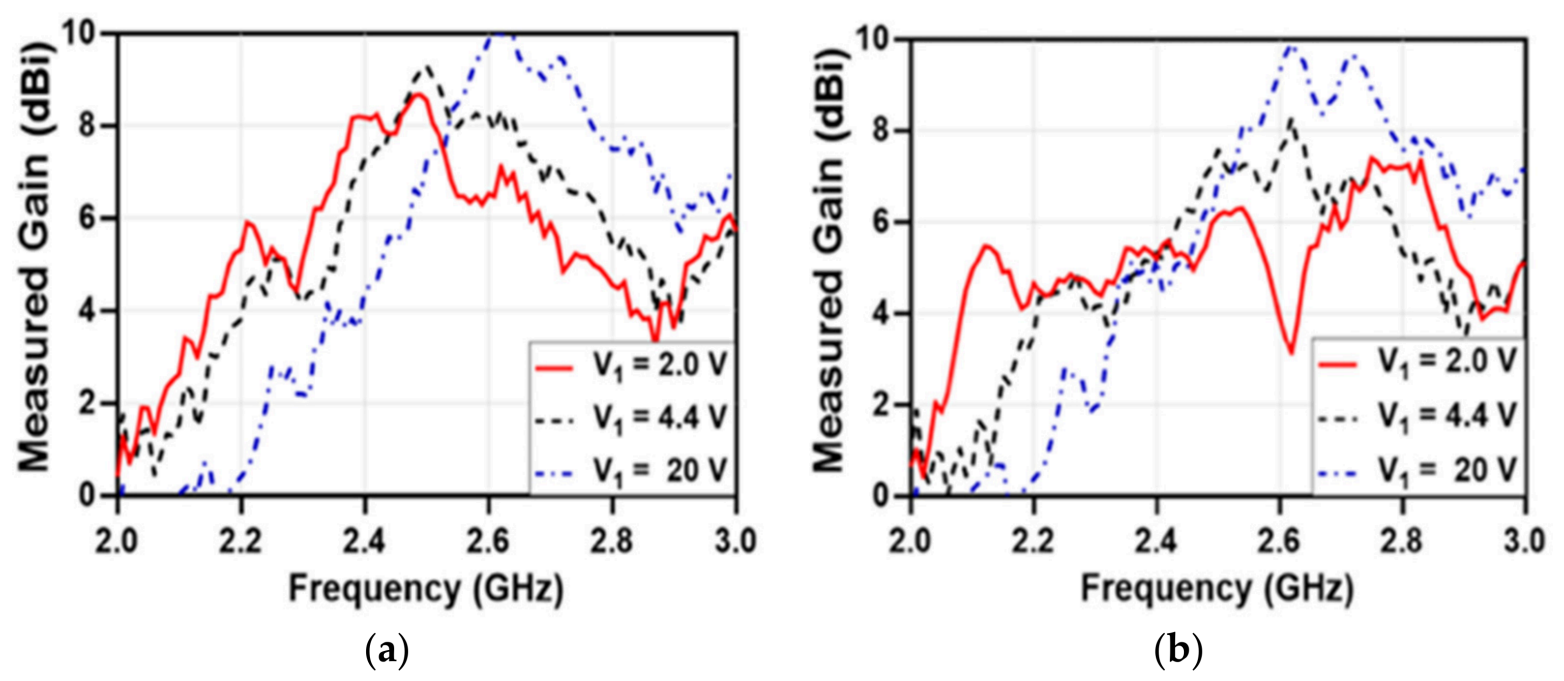
Measured gain for different applied voltages of the driven elements: (**a**) reflector mode; (**b**) director mode.

Figure 8 shows the measured peak-realized gain for reflector and director modes at different driven-element varactor capacitances. It can be observed that with C_1_ tuning, the antenna maintains peak gain between 6 and 10 dB for both reflector and director modes across the operating band.

### 3.3. MIMO Performance

**Figure 9:**
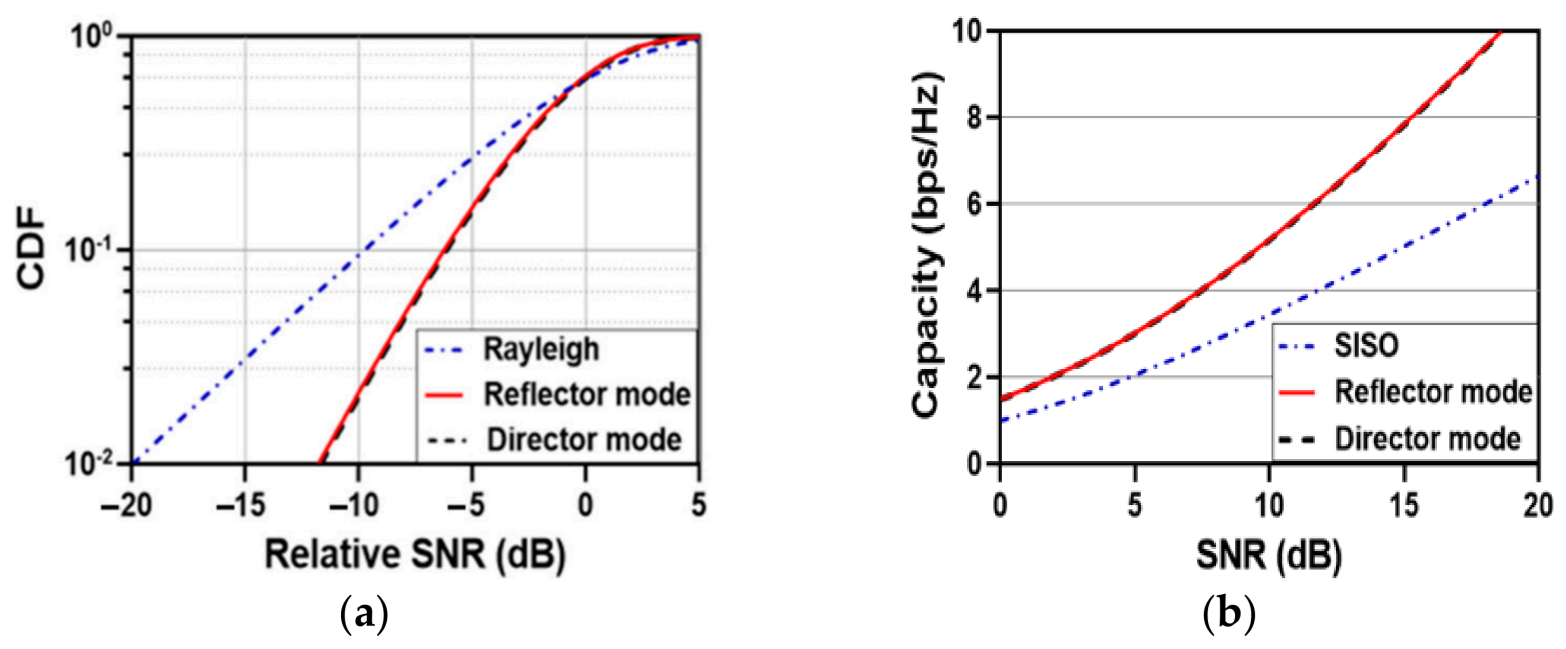
Diversity and MIMO performance of the proposed antenna at 2.4 GHz: (**a**) CDF of relative SNR; (**b**) MIMO capacity.

The diversity and MIMO performance of the proposed antenna were analyzed to evaluate the system-level performance. Figure 9a shows the Cumulative Density Function (CDF) [31] of relative Signal-to-Noise Ratio (SNR) [32] of the proposed antenna for reflector and director modes along with Rayleigh distribution (as a reference). The results indicate that both modes exhibit nearly identical performance with good diversity characteristics. The calculated Effective Diversity Gain (EDG) [23] is 8.2 dB at a 1% outage probability. 

The ergodic capacity of a MIMO system employing N transmit and receive antennas can be expressed as [31,33]:

(3) $$C=E\{{log}_{2}[det(I_{N}+\frac{\gamma {.e}_{emb}}{N}\Phi HH^{H})]\}$$

where E{.} and det(.) represent the expectation and the determinant operators, respectively, $I_{N}$ denotes the N × N identity matrix, γ is the Signal-to-Noise Ratio, and $e_{emb}$ represents to the embedded efficiency, respectively. H is the channel matrix, while superscript H denotes the Hermitian (complex conjugate transpose). Φ is the spatial correlation matrix that is given for the two-antenna configuration, which is given as:

(4) $$\Phi =\left[\begin{array}{cc} 1 & \rho_{c12}\\ \rho_{c12}^{*} & 1 \end{array}\right]$$

where $\rho_{cij}$ denotes the complex cross-correlation coefficient between the ith and jth antenna elements, defined as:

(5) $$\rho_{cij}=\frac{\int_{0}^{2\pi }\int_{0}^{\pi }A_{ij}(\Omega )d\Omega }{\sqrt{\int_{0}^{2\pi }\int_{0}^{\pi }A_{ii}\left(\Omega \right)d\Omega .\int_{0}^{2\pi }\int_{0}^{\pi }A_{jj}(\Omega )d\Omega }}$$

where Ω ≡ (*θ*, Φ) denotes the angular coordinates, and *d*Ω ≡ sin*θdθd*Φ. The term $A_{ij}(\Omega )$ is expressed as:

(6) $$A_{ij}=XPRE_{\theta ,i}\left(\Omega \right)E_{\theta ,j}^{*}\left(\Omega \right)P_{\theta }\left(\Omega \right)+E_{\Phi ,i}\left(\Omega \right)E_{\Phi ,j}^{*}(\Omega )P_{\phi }\left(\Omega \right)$$

where $E_{\theta }\left(\Omega \right)$ and $E_{\phi }\left(\Omega \right)$ are the θ and ϕ polarized far-field components of the antenna gain pattern, respectively, and XPR is the cross-polarization power ratio. $P_{\theta }\left(\Omega \right)$ and $P_{\phi }\left(\Omega \right)$ corresponds to the angular power distributions for the respective polarizations.

Table 3 summarizes the key MIMO performance metrics of the proposed antenna across three operating frequencies—2.4 GHz (low band), 2.6 GHz (mid band), and 2.8 GHz (high band)—evaluated in both reflector and director modes. The design demonstrates strong overall radiation efficiency, ranging from 77.0% at the lowest frequency up to a peak of 92.4% in the mid-band director mode, alongside stable peak gains spanning 4.02 dBi to 5.27 dBi across all configurations. Notably, the antenna exhibits exceptional diversity performance characterized by exceedingly low Envelope Correlation Coefficient (ECC) values, which remain well below standard acceptable thresholds and drop to as low as 0.00002 in the high-band reflector mode. Consequently, the system yields near-ideal Diversity Gain (DG) values approaching the theoretical maximum of 10 dB across all evaluated bands. Furthermore, the Mean Effective Gain (MEG) remains consistent between 0.39 and 0.46, and the multiplexing efficiency operates favorably between −1.17 dB and −0.33 dB, collectively validating the proposed design’s suitability for high-performance, low-correlation MIMO communication systems. 

For channel modeling, a uniform propagation environment is assumed, which is a valid approximation for rich-scattering indoor and outdoor scenarios where MIMO systems are typically deployed. The ergodic MIMO capacity of the proposed antenna, along with that of a single-input single-output (SISO) reference, was computed in MATLAB R2021b (Version 9.11) using 50,000 channel realizations. The results, illustrated in Figure 9b, confirm that the proposed antenna achieves a substantially higher capacity than the SISO configuration for both reflector and director modes.

## 4. State-of-the-Art Comparison

Table 4 highlights the advantages of the proposed antenna by contrasting it with existing frequency- and pattern-reconfigurable models. Conventional approaches frequently rely on intricate multi-layer arrangements or parasitic geometries requiring extensive switching networks, classified as complex in Table 4. References [34,35,36,37,38,39], for example, incorporate between 6 and 36 PIN diodes or varactors to achieve pattern and frequency agility. While these methods succeed functionally, they inherently increase DC biasing demands and the overall footprint, rendering them impractical for integration into modern compact electronics.

Conversely, the introduced geometry utilizes a straightforward two-element printed dipole. By integrating just one parasitic element per dipole, the entire structure occupies a minimal 120 × 22 mm^2^ area on a standard 0.8 mm FR4 board. Both frequency tuning and radiation pattern shifting are accomplished using a total of four varactor diodes. This drastic reduction in active components significantly lowers the control complexity compared to the surveyed literature.

A major distinction of this work is its built-in MIMO capability, a feature largely absent in contemporary reconfigurable designs. Although earlier studies feature array setups, they generally rely on shared feed networks rather than isolated ports dedicated to spatial multiplexing or diversity. The 2 × 2 MIMO configuration presented here delivers simultaneous pattern and frequency adaptability, equipping modern handheld terminals to better handle dynamic multipath environments.

Evaluating the operational metrics, the antenna achieves a 24% frequency-tuning span (from 2.2 to 2.8 GHz). This outperforms most of the benchmarks in Table 4, which generally peak at a 20% fractional bandwidth. The design also supports dual-beam steering at 170° and 350° in the azimuth, ensuring the orthogonal spatial coverage required for robust portable MIMO systems. Throughout these tuning states, the prototype maintains stable characteristics, yielding measured radiation efficiencies from 58% to 89% and peak gains between 4.8 and 10 dBi.

Ultimately, this dipole-based architecture sets itself apart by successfully uniting structural simplicity, a small footprint, and dual-parameter tuning with true spatial multiplexing. Co-integrating these capabilities remains a challenge in the recent literature, underscoring the practical viability and originality of this antenna for upcoming wireless platforms.

## 5. Conclusions

In this paper, a two-element MIMO antenna with frequency and pattern reconfiguration capabilities for mobile handsets is presented. The antenna employs varactor diodes loaded on both the driven and parasitic dipole elements as the reconfiguration mechanism. By tuning the effective length of the driven dipoles, three distinct narrow bands between 2.2 and 2.8 GHz were achieved and reconfigured. Two different beam directions, at 350° and 170° in the azimuth plane were obtained at all frequency bands by tuning the length of the parasitic dipoles. The measurement results show good agreement with the simulations validating the design concept. Compared to previously reported reconfigurable antennas, the proposed design demonstrates a simpler configuration that simultaneously achieves wide-frequency tunability and dual-direction beam steering while maintaining compact size, low cost, and high efficiency, without relying on complex switching networks or multiple control circuits. Thus, the proposed antenna has strong potential to be employed in smart and cognitive radio applications.

## Figures and Tables

**Table 1:** Varactors capacitance in simulation and corresponding control voltage in measurement for frequency and pattern tuning.

Band	Mode	Simulation		Measurement	
		C1 (pF)	C2 (pF)	V1 (V)	V2 (V)
Band 1 (low band)	Reflector	1.0	1.0	2.0	2.0
	Director	1.0	0.1	2.0	22
Band 2 (mid band)	Reflector	0.5	0.8	4.4	4.7
	Director	0.5	0.1	4.4	22
Band 3 (high band)	Reflector	0.2	0.7	20	10.3
	Director	0.2	0.1	20	22

**Table 2:** Measured highest coupling coefficient (S_21_) between the antenna elements at different frequency bands and pattern modes.

Band	Mode	Voltage		Measured S21 (dB)
		V1 (V)	V2 (V)	(Worst)
Band 1 (low band)	Reflector	2.0	2.0	−17.6
	Director	2.0	22	−16.7
Band 2 (mid band)	Reflector	4.4	4.7	−16.7
	Director	4.4	22	−17.9
Band 3 (high band)	Reflector	20	10.3	−15.7
	Director	20	22	−16.0

**Table 3:** MIMO Parameters of the proposed antenna for different bands and modes.

Band	Band 1 (Low Band) 2.4 GHz		Band 2 (Mid Band) 2.6 GHz		Band 3 (High Band) 2.8 GHz	
Mode	Reflector	Director	Reflector	Director	Reflector	Director
Efficiency (%)	77.0	91.3	86.7	92.4	92.3	87.5
Peak Gain (dBi)	4.93	4.02	4.92	5.05	4.79	5.27
Mean Effective Gain (MEG)	0.39	0.46	0.43	0.46	0.46	0.45
Envelope Correlation Coefficient (ECC)	0.014	0.0006	0.0001	0.0002	0.00002	0.002
Diversity Gain (DG) (dB)	9.97	10.0	10.0	9.999	10.0	9.99
Multiplexing Efficiency (dB)	−1.17	−0.39	−0.63	−0.34	−0.33	−0.59

**Table 4:** Comparison of proposed antenna performance with relevant frequency- and pattern-reconfigurable antenna designs in the literature.

Ref.	Antenna Structure (Complexity)	Size (mm^2^) /Substrate	Reconfiguration (Actuators)	Operation Mode/ Active Elements	Bandwidth (GHz)/BW%/ Steering Angles	Efficiency (%)/ Peak Gain (dBi) (Simulated or measured)	Mutual Coupling /ECC/ Applications
[9]	Stub-loaded patch array (1 × 2) (Simple)	151.5 × 160.9 /Duroid 5880 1.6 mm	Frequency and pattern (2 Varactors)	Array / 2 elements	(2.15–2.38) GHz /10%/ −23° to +23°	Mea. (40–85) % / (4.1–9.0) dBi	Not Provided/ Two-element tunable array
[11]	Monopole with switchable diplexer (Complex)	120 × 143 /Rogers Diclad 880 0.76 mm	Frequency and pattern (4 Varactors)	Array / 2 elements	(2.24–2.52, (5.68–5.87) GHz /11.5%, 3.8%/ bidirectional	Mea. (75–91) % (1.4–7.7) dBi	−20 dB/NA/ Dual-band bidirectional radiation
[34]	Center-shorted stub-loaded microstrip patch (Complex)	53 × 18 /Duroid 5880 1.6 mm	Frequency and pattern (6 Varactors)	Single Element	(2.68–3.51) GHz /20%/ broadside to omni	Mea. (40–82) % / (1.1–7.8) dBi	NA/ Dual-mode smart communications
[35]	Double-layer petal-shaped parasitic structure (Complex)	100 × 100 /F4B 1 mm	Frequency and pattern (28 PIN diodes + 8 Varactors)	Single Element	(2.20–2.52) GHz /13.6%/ 0° to 360°	Sim. (83–88) % / (1.5–5.6) dBi	NA/ Hybrid Reconfigurable applications
[36]	Slot with reflector and reconf. ground slits (Complex)	130 × 160 /Taconic RF35 1.52 mm	Frequency and pattern (14 PIN diodes)	Single Element	(1.75–2.20) GHz /22.7%/ −15° to +15°	Sim. (86–90) % / (3.2–6.3) dBi	NA/ Cognitive radio applications
[37]	Circular disk loaded with slots (Complex)	40 × 60 /FR4 1.6 mm	Frequency and pattern (15 PIN diodes)	Single Element	(2.3–2.5, 5.1–5.9) GHz /8.3%, 14.5%	Not Provided / Sim. (2–5.8) dBi	NA/ WiMAX, Wi-Fi, WLAN
[38]	Segmented patch antenna (Complex)	50 × 50 /Duroid 5880 1.6 mm	Frequency and pattern (5 PIN diodes)	Single Element	(4.1–5.7) GHz /45%/ 0° to 360°	Mea. (42–92) % / (1.7–4.2) dBi	NA/ Internet of Vehicles
[39]	Microstrip patch array (Complex)	200 × 83 /F4B (3 mm)	Frequency and pattern (18 PIN + 12 Var)	Array / 4 elements	(3.54–4.46) GHz /23%/ −55° to +55°	Mea. (12–44) % / (0.7–7.7) dBi	Not Provided/ Four-element Array Tunable array
[40]	Printed dipole (Simple)	105 × 60 /FR4 (1.5 mm)	Frequency and pattern (4 PIN Diodes)	MIMO / 2 elements	(2.2–2.8) GHz /23%/ −5° to 5°	Mea. (62–75) % / Not provided	−15 dB /Not Provided/ MIMO
[41]	Printed dipole (Complex)	62.3 × 48 /TLY-5 (1.17 mm)	Frequency and pattern (2 Photo Switches)	Single Element	(2.2–2.3) GHz, (2.95–3.35) GHz /9.5%, 13.4%/ 0° to 19°	Not provided / (2.9–4.0) dBi	NA/ Optically Reconfigurable dual band
[42]	Meandered patch (Complex)	162.6 × 77.7 /Duroid5880 (0.508 mm)	Frequency (9 PIN Diodes)	MIMO 9 elements	(3.45–3.55) GHz, (27.5–28.5) GHz /7.4%, 4.7%	Mea. 93.6% / (2.3–7.7) dBi	−10 dB/0.11/ Sub-6 GHz and mm-wave
[43]	Meandered monopole (Simple)	33 × 16 /FR4 (1.6 mm)	Frequency (3 PIN Diodes)	Single Element	(2.1–5.2) GHz, /(9.35–41.6) %	Mea. (80.4–96) % / (1.85–3.46) dBi	NA/ Hexa-band, UMTS, Wi-MAX
[44]	Slot array (Simple)	150 × 100 /RO3003 (0.13 mm)	Pattern (4 Phase shifters)	MIMO 8 elements	(27.4–28.23) GHz, /135°, 155°, 205°, 230°	Not provided / Mea. 4.3–6.6 dBi	−9 dB/0.009/ mm-wave and 5 G
This Work	Dipole with parasitic element (Simple)	120 × 22 /FR4 0.8 mm	Frequency and pattern (4 Varactors)	MIMO 2 elements	(2.2–2.8) GHz /24%/ 170° and 350°	Mea. (58–89) % / 4.8–10 dBi	−15.7 dB/0.014/ compact devices

## Data Availability

The datasets used and/or analyzed during the current study are available from the corresponding author on reasonable request.
